# Associations Between Positive Affect and Heart Rate Variability: A Systematic Review

**DOI:** 10.1007/s11886-025-02299-4

**Published:** 2025-10-23

**Authors:** Martha Schneider, Christian Rominger, Andreas R. Schwerdtfeger

**Affiliations:** University of Graz, Strassolodogasse 10, Graz, 8010 Austria

**Keywords:** Positive affect, Heart rate variability, Arousal, Systematic review

## Abstract

**Purpose of review:**

The relationship between positive affect (PA) and heart rate variability (HRV) has attracted considerable interest due to its potential implications for emotion regulation and cardiovascular health. This systematic review synthesizes current literature on the association between PA and HRV, considering resting-state, stress-reactivity and recovery contexts, as well as variations in PA conceptualization.

**Recent findings:**

A total of 36 studies (*N* = 5501) were included, spanning experimental, ambulatory, cross-sectional, and mixed designs. Elevated PA was most often associated with higher vagally mediated HRV, measured as RMSSD or high-frequency (HF) power, but results varied by context. Resting-state and trait-like PA measures showed the most consistent positive associations. In stress-induction paradigms, effects depended on the stress phase and arousal level of PA, with RMSSD emerging as a more consistent index than other HRV metrics. In real-life settings, aggregated activated PA was linked to higher RMSSD, while momentary activated PA was linked to lower RMSSD, suggesting short-term allostatic adjustments. Findings for other HRV metrics, such as LF/HF ratio, LF-HRV, and SDNN, were mixed.

**Summary:**

Overall, this review highlights the complex interplay between PA and cardiac autonomic regulation and provides directions for future research, which should aim for greater methodological consistency and clarify the temporal dynamics of the PA-HRV relationship.

## Introduction

The interconnection between emotional well-being and cardiovascular health has been widely discussed [1–3]. Prospective epidemiological studies suggest that positive emotions, such as compassion and happiness may exert a protective effect on cardiac health [3, 4]. In this context, positive affect (PA) – the experience of pleasurable emotions such as joy, enthusiasm, and contentment - emerges as a crucial psychological construct. In a large, population-based study, Davidson et al. [5] showed that increased PA was protective against 10-year incident coronary heart disease (CHD). Furthermore, Hoen et al. [6] found in a sample of outpatients with CHD that PA was associated with improved survival. Importantly, research indicates that protective effects of PA on cardiac health may stem from beneficial autonomic cardiac regulation [7–9].

Autonomic cardiac control can be examined through heart rate variability (HRV). HRV refers to the variations in time between successive heart beats. It reflects autonomic nervous system (ANS) flexibility, meaning the dynamic interplay between the sympathetic and parasympathetic nervous system [10]. Time-domain measures, such as the standard deviation of NN intervals (SDNN) and the root mean square of successive differences (RMSSD) provide insight into overall autonomic regulation, with RMSSD being particularly sensitive to parasympathetic (i.e., vagal) activity. Frequency-domain analyses of HRV are based on the spectral decomposition of interbeat intervals. High-frequency (HF) power, ranging from 0.15 to 0.40 Hz, is closely associated with parasympathetic modulation, while low-frequency (LF) power, spanning from 0.04 to 0.15 Hz, seems to be influenced by both sympathetic and parasympathetic processes [11]. The LF/HF ratio has commonly been used to estimate the balance between sympathetic and parasympathetic activity, though its interpretation is subject to debate [12, 13].

Elevated HRV has been associated with enhanced cardiovascular adaptability and lower lifetime risk of cardiovascular disease (CVD). For instance, a meta-analysis by Hillebrand et al. [9] found that in individuals without a known cardiovascular disease, higher HRV is linked to a lower risk of first-time cardiovascular event. Additionally, Tsuji et al. [14] demonstrated that individuals with higher resting HRV had a lower risk of cardiac mortality, indicating better cardiovascular adaptability. In contrast, diminished HRV is linked to a higher risk of an initial cardiovascular event [9] and accelerated atherosclerosis progression. Supporting this observation, a prospective cohort study by Huang et al. [15] demonstrated that individuals with lower resting HRV exhibited greater progression of carotid atherosclerosis over time.

Theoretical considerations on the relationship between PA and HRV focus on different mechanisms, through which PA and HRV could mutually influence each other. Several models propose that positive affect influences HRV. The broaden-and-built theory of positive emotions [16] suggests that PA broadens cognitive and behavioural repertoires, which in turn improves coping, emotional regulation, and stress recovery, thereby promoting greater autonomic flexibility—a physiological capacity closely linked to higher HRV. In line with this assumption, studies suggest that PA may enhance autonomic flexibility by reducing physiological stress responses and increasing parasympathetic activity, which in turn is reflected in elevated HRV [6, 17]. Experimental studies have shown that eliciting PA can induce immediate increases in HRV, indicating that even brief episodes of PA may promote parasympathetic dominance [18].

Other theoretical accounts reverse this causal direction, proposing that HRV influences PA. The Neurovisceral Integration Model [19] highlights, that HRV reflects prefrontal cortex control over the ANS, thereby promoting effective emotional regulation and the facilitation of positive emotional experiences. Higher HRV may therefore be associated with more frequent experiences of PA, due to greater self-regulation capacity and stress adaptivity. Together, these perspectives suggest a bidirectional relationship, in which PA can produce short-term parasympathetic activation and potentially contribute to sustained autonomic flexibility, while higher HRV can foster emotional regulation and thereby increase the likelihood of positive affect over time.

Building on these theoretical considerations, it is essential to recognize that PA is not a unified construct but can be conceptualized through various facets. PA can be defined as a stable, enduring disposition (trait PA), or as a momentary, situational manifestation (state PA). Furthermore, trait PA seems to reflect a stable tendency towards experiencing positive emotions and is more consistently linked to long-term health benefits such as improved cardiovascular adaptability and autonomic regulation [20]. In contrast, relations between state PA and health tend to be less stable and more dependent on situational contexts [21]. Additionally, a distinction based on the activation level of PA can be applied [22]. Specifically, activated PA refers to high-arousal, energetic positive emotions, such as excitement, enthusiasm and elation. In contrast, deactivated PA encompasses low-arousal, calm states, like contentment and relaxation.

In view of these conceptual variations, the empirical literature on the relationship between PA and HRV is marked by considerable heterogeneity. Furthermore, confounding variables may play an important role in influencing the PA-HRV relation. Demographic variables such as age and gender [23], as well as physical activity [24], or medication use (e.g., beta blockers [25]) may modulate autonomic function and therefore influence the PA-HRV relationship. Accounting for these factors seems crucial for understanding the relation between PA and HRV, and to ensure that the observed effects are not biased by external factors. Considering the manifold influences on the relationship between PA and HRV, it becomes evident that a systematic review is essential to synthesize findings and clarify their multifaceted interactions. Hence, the aim of the following review was to systematically evaluate the relationship between PA an HRV by critically evaluating study methodologies, outcomes, and moderating factors. To ensure comprehensiveness, the present review considered studies assessing HRV at rest and in response to or recovery from stress. This approach aligns with the Vagal Tank Theory [26]), which emphasizes that HRV should be understood dynamically across different contexts, including resting state, stress reactivity, and recovery. Each of these provides unique and complementary insights into self-regulatory capacity. Considering HRV across these contexts provides a more comprehensive basis for examining the PA-HRV association. Therefore, this review seeks to clarify inconsistencies and guide future research directions in understanding interrelations between PA, cardiac autonomic regulation and cardiovascular health.

## Methods

The review was conducted using PRISMA (Preferred Reporting Items for Systematic Reviews and Meta-Analysis) guidelines [27]. Four databases were screened for eligible articles, Scopus, PsycInfo, PubMed and Web of Science. The database search was conducted from 8th to 13th of January 2025. The search was not limited to any specific language. The following search string was used in all databases searched: (positive affect’) AND (‘heart rate variability’ OR ‘heart rate variabilities’ OR ‘HRV’ OR ‘HR variability’ OR ‘HR variabilities’).

**Fig. 1:**
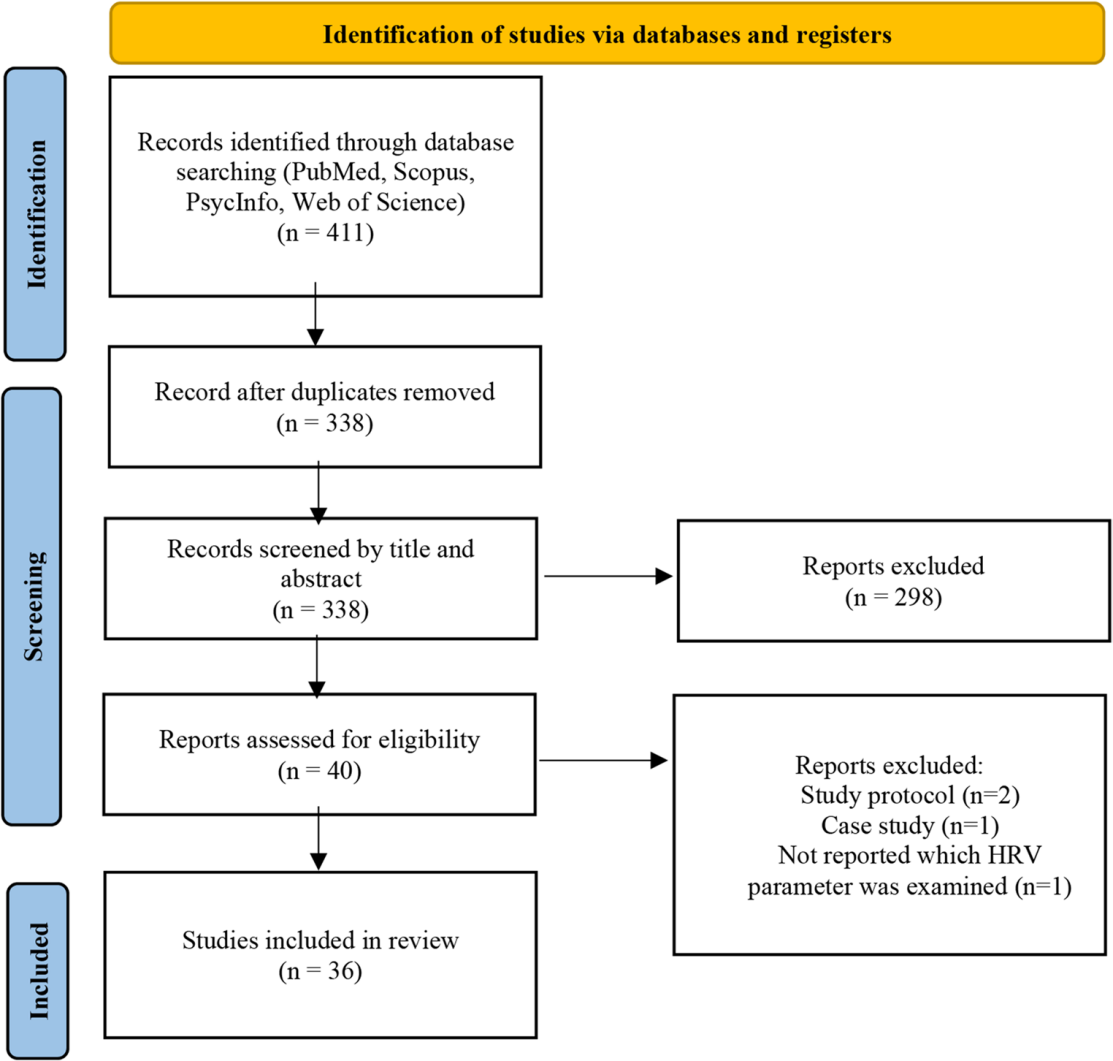
Study selection process

Included studies needed to report data on HRV metrics and PA measures. Conference papers as well as case studies were excluded. In the first step, titles and abstracts were screened for compatibility. After that, duplicates were removed. For the remaining articles the full text was screened. To be included, a study had to (1) report a measure of PA or a PA induction task and (2) report measures of HRV with clear indication which HRV parameters were examined. Resting-state HRV, HRV reactivity to stress, and HRV recovery following stress were included to represent both baseline autonomic activity and the capacity to regulate physiological responses to environmental demands. The following information was taken from the studies: (1) author (2) year of publication, (3) sample size, age and gender of participants (4) study design (5) PA type (6) PA measurement (7) HRV Domain (8) Main Findings. All HRV metrics were considered eligible for inclusion, with the aim of reflecting the diversity of operationalizations found in the literature. No HRV metrics were excluded a priori. Figure 1 shows details of the search process.

## Results

### Overview of the Search Process

A total of 411 articles were identified through database search (129 from Scopus, 96 from PsycInfo, 80 from PubMed, 106 from Web of Science). After removing duplicates and screening titles and abstracts, 40 potentially applicable articles remained for full-text screening (see Fig. 1). Four articles were removed (two study protocols, one case study and one study, which did not provide details on which HRV parameters were examined). In total, 36 studies were included in the current review.

### Description of Included Studies

The 36 studies involved information on 5,501 individuals. The average age of participants across all studies ranged from 9 to 82 years. Most studies (*n* = 29, 80.56%) reported a higher female to male ratio. Most studies used experimental study designs (*n* = 11, 30.55%) or ambulatory study designs (*n* = 9, 25%), followed by cross-sectional studies (*n* = 8, 22.21%), and mixed study designs, combining cross-sectional and ambulatory designs (*n* = 7, 19.44%). One study (*n* = 1, 2.8%) was based on a longitudinal study design.

State PA was examined in 25 studies (69.44%), seven studies focused on trait PA (19.44%), two studies examined both state and trait PA (5.56%), two studies did not report PA type (5.56%). Most studies (*n* = 21, 58.33%) used the Positive and Negative Affect Schedule (PANAS) [65] as a PA measure. All HRV metric reported in the included studies are summarized in Table 1. The most frequently examined metrics were RMSSD (*n* = 21) and HF-HRV (*n* = 19), followed by LF-HRV (*n* = 7) and LF/HF ratio (*n* = 6). Four studies examined SDNN, one study SDANN, three studies focused on RSA, two studies included VLF. One study focused on a wider range of HRV metrics, including pNN50,* TP*,* SD1*,* SD2 and SD2/SD1.* Age and gender emerged as the most frequently controlled confounding variables in the analyses. Table 2 provides an overview of the key characteristics of the included studies.

**Table 1:** Overview of heart rate variability (HRV) metrics reported across included studies

Abbreviation	Full term
RMSSD	Root Mean Square of Successive Differences
SDNN	Standard Deviation of NN intervals
SDANN	Standard Deviation of the Average NN intervals
pNN50	Percentage of NN intervals differing >50 ms
HF-HRV	High-Frequency HRV (0.15-0.4 Hz)
LF-HRV	Low-Frequency HRV (0.04-0.15 Hz)
LF/HF	LF/HF Ratio
VLF	Very Low Frequency (<0.04 Hz)
RSA	Respiratory Sinus Arrhythmia
TP	Total Power
SD1, SD2	Poincaré plots
SD2/SD1 ratio	Ratio of SD2/SD1

**Table 2:** Characteristics of included studies

Author & Year	Sample (*N*, Age, Gender)	Study design	PA Assessment	PA Measurement	HRV Domain	Main Findings
Acevedo et al., 2022 [28]	238 adults (*M* = 20.6, 75.6% female)	Experimental (between)	State	PA inducing task	RMSSD	*Low arousal calm and high arousal excited PA showed steeper increase in RMSSD from post induction to stress task compared to neutral condition*
Beatton et al., 2024 [29]	321 adults (*M* = 43.2, 40.2% female)	Ambulatory (within)	State	Mood rating, one-item	LF/HF RMSSD	*PA was negatively related to higher LF/HF during an activity; PA was positively related to RMSSD*
Bhattacharyya et al., 2008 [30]	76 patients with suspected CAD (*M* = 61.1, 31.6% female)	Ambulatory (between)	State	Day reconstruction method	HF LF	High PA was associated with greater HF and lower LF
Bylsma et al., 2024 [31]	303 adults (M = 33.9, 68% female)	Mixed (within & between)	Trait	Four-item measure	HF RMSSD	No significant relation between PA, PA variability and RMSSD or HF
Conrad et al., 2008 [32]	46 depressed and 19 non-depressed adults (61.1–64.3, 60% female)	Ambulatory (within & between)	State	PANAS	HF LF VLF TF-RSA,	No significant relation between PA and HF, LF, VLF or TF-RSA
Duarte et al., 2017 [33]	91 adults (M = 20.5, 100% female)	Cross-sectional (between)	Trait	ToPAS & PANAS	HF RMSSD	*Significant quadratic relationship (inverted U-shape) between PA and HF*
Fernandes et al., 2017 [34]	50 patients with MDD (M = 45.6, 64% female)	Cross-sectional (between)	n.r.	PANAS	HF LF RMSSD	No significant relation between PA and HF, LF or RMSSD
Gouin et al., 2019 [35]	149 adults (M = 21.8, 100% female)	Mixed (within & between)	State	PANAS (4 selected items)	RSA	*Lower PA was associated with higher RSA augmentation towards a worry induction task*
Hachenberger et al., 2023 [36]	26 adults (M = 23.8, 88.46 female)	Ambulatory (within & between)	State	PANAS & Russell’s Circumplex Model of Affect (3 selected items)	LF HF LF/HF RMSSD	*PA was positively associated with LF/HF ratio*,* PA was negatively associated with HF and RMSSD*
Kochli-Hailovski et al., 2021 [37]	104 adults (M = 67.3, 76.9% female)	Cross-sectional (between)	Trait	Brief PANAS	HF RMSSD SDNN	*Lower PA was associated with lower HRV (latent variable composed of SDNN and RMSSD)*
Kop et al., 2011 [38]	20 adults (M = 25, 55% female)	Experimental (within)	State	PA inducing task	HF LF LF/HF	Positive association between PA and HF
Koval et al., 2013 [39]	83 adults (M = 19, 62.7% female)	Mixed (within & between)	State	PA & PA instability (6 selected items)	HF RMSSD	*Instability of PA was negatively related to HF & RMSSD*
Lane et al., 2011 [40]	161 Long QT syndrome patients (M = 35, 71% female)	Ambulatory (within)	State	PANAS (16 selected items)	HF	Negative associations between activated PA and HF, positive associations between low arousal PA and HF
Määttänen et al., 2021 [41]	44 adults (M = 25, 77.3% female)	Ambulatory (within & between)	State	3-item measure (adapted from PANAS)	SDNN	Higher PA associated with higher SDNN
Martinez et al., 2022 [42]	657 adults (M = 35.2, 40.5% female)	Ambulatory (within)	State	PANAS	SDNN RMSSD SDANN pNN50 LF/HF TP VLF SD1 SD2 SD2/SD1	Higher PA was associated with higher VLF, lower PA was associated with higher LF/HF and SDANN.”
Matos et al., 2022 [43]	93 adults (M = 23.3, 90.3% female)	Longitudinal (between)	Trait	ToPAS	RMSSD	Relaxed PA was enhanced by the intervention, an effect mediated by increases in RMSSD.”
Michels et al., 2022 [44]	92 adults (18–30, 83.7%% female)	Experimental (between)	State	POMS PANAS	RMSSD	No significant relation between PA and RMSSD
Moreno et al., 2023 [45]	124 adults (M = 37.4, 100% male)	Experimental (within & between)	State	PANAS	RMSSD	No significant relation between PA and RMSSD
Movahed et al., 2017 [46]	99 children (9–11 years, 51.5%female)	Experimental (within & between)	State	PANAS (child version)	HF	PA and HF at preparation were positively correlated
Noah et al., 2015 [47]	18 adults (Group 1 M = 25.1, 10% female, Group 2 M = 31.2, 25% female)	Experimental (between)	State	I-PANAS-SF	LF HF LF/HF	Positive relation between PA and LF/HF
Papousek et al., 2010 [48]	65 adults (M = 20.5, 81.5% female)	Experimental (within)	State & Trait	PANAS Single item measure	LF HF LF/HF	Higher trait PA was associated with more efficient recovery of LF/HF, higher state PA associated with less efficient recovery of LF/HF and LF
Petrocchi et al., 2017 [49]	34 adults (M = 43.7, 58.8% female)	Experimental (within)	State	ToPAS	HF SDNN	Significant associations between soothing PA (delta score) and HF (delta score)
Pinto et al., 2022 [50]	187 adults (M = 82.1, 80.7% female)	Cross-sectional (between)	State	PANAS	HF	No relation between PA and HF
Rost et al., 2021 [51]	46 patients with fibromyalgia, 46 healthy controls (Group 1 F = 45.4, 84.8% female, Group 2 = 44.9, 80.4% female)	Mixed (within & between)	State	Six-item measure, PA instability	RMSSD	No relation between PA instability and RMSSD
Schilling et al., 2020 [52]	201 adults (M = 38.6, 35.8% female)	Mixed (within & between)	State	PANAS (5 selected items)	RMSSD	No relation between PA and RMSSD
Schwerdtfeger et al., 2014 [53]	117 adults (M = 27.8, 57% female)	Ambulatory (within & between)	State & Trait	6-item measure (adapted from PANAS)	RMSSD	Aggregated activated PA and momentary deactivated PA was associated with higher RMSSD, activated momentary PA was accompanied by lower RMSSD,
Schwerdtfeger et al., (2015) [54]	63 adults (M = 28.8, 50.8% female)	Ambulatory (between)	State	9-item measure	RMSSD	Aggregated deactivated PA during the day was associated with higher nocturnal RMSSD
Shell et al., 2022 [55]	216 patients with depression (M = 58.7, 78.2% female)	Cross-sectional (between)	Trait	PANAS	HF	No relation between PA and HF
Sloan et al., 2016 [56]	967 adults (M = 54.6, 100% female)	Cross-sectional (between)	n.r.	PANAS & MASQ	HF	No relation between PA and HF
Spangler et al., 2021 [57]	305 adults (M = 19.9, 58.7% female)	Cross-sectional (between)	Trait	PANAS	HF RMSSD	Significant positive linear relation between RMSSD and PA in men, inverted-U relation between RMSSD and PA in women
Sveinsdóttir et al., 2023 [58]	54 adults (M = 27.7, 53% female)	Experimental (within & between)	Trait	PANAS	RMSSD	Higher RMSSD associated with higher levels of PA
van der Ploeg et al., 2016 [59]	18 adults (M = 21, 62.1% female)	Experimental (within & between)	State	IPANAT	RMSSD	No relation between Explicit or Implicit PA and RMSSD
Verkuli et al., 2016 [60]	32 adults (M = 21.1, 75% female)	Mixed (within & between)	State	Single-item measure	RMSSD	*Lower explicit PA was associated with more frequent additional reductions in RMSSD (AddHRV)*,* defined as values exceeding two SE below expected HRV levels*
Wang et al., 2013 [61]	98 adults (M = 20, 78.6% female)	Mixed (within & between)	Trait	PANAS	RSA	Positive relation between PA and resting RSA
Weiss et al., 2021 [62]	122 adults (M = 23.4, 84.4% female)	Cross-sectional (between)	State	PANAS	HF	Positive relation between positive emotion dysregulation and resting HF at high state PA and negative relation between positive emotion dysregulation and resting HF at low state PA
Weyn et al., 2022 [63]	101 adolescents (M = 11.6, 54.6% female)	Experimental (within & between)	State	PANAS	RMSSD	No significant relation between PA and RMSSD

M, mean; HF, high-frequency band, LF, low-frequency band, VLF, very low frequency band, RMSSD, root mean square of successive RR interval differences; SDNN, standard deviation of NN intervals, LF/HF, ratio of LF-to-HF power; TP, total power; RSA, respiratory sinus arrhythmia, TF-RSA, transfer function respiratory sinus arrhythmia, SDANN; standard deviation of the 5 min average NN intervals; pNN50, proportion of NN50 divided by total number of NN intervals, AddHRV, Additional reductions in HRV referring to time points where RMSSD was more than two standard errors below the expected RMSSD level, as defined by individual calibration models, reflecting non-metabolic reductions in HRV that are independent from physical activity. PANAS, Positive and Negative Affect Schedule [64], IPANAT Implicit Positive and Negative Affect Test [65], POMS, Profile of Mood State [66], ToPAS, Typoes of Positive Affect Scale [67], MASQ, Mood and Anxiety Symptom Questionnaire [68], Circumplex Model of Affect [69]

### PA-HRV Relations in Stress Contexts

Fourteen studies examined the relationship between PA and HRV under stress conditions, employing both laboratory-induced stressors and real-life assessments of perceived stress. Experimental laboratory studies assessing the entire stress induction period show mixed findings regarding the association between PA and HRV. Sveinsdóttir et al. [58] found that individuals with high trait PA exhibited elevated RMSSD during various stress tasks, while Noah et al. [47] observed that a gaming paradigm eliciting PA was associated with a higher LF/HF ratio.

Differences in the relationship between PA and HRV are also evident upon examining the distinct phases of stress paradigms. During the preparation phase preceding a stressor, HRV appears to be positively correlated with PA. Movahed et al. [46] used a social stress task and observed a positive correlation between state PA and HF-HRV at rest. Similarly, Wang et al. [61], using an emotion-based stress task, reported that trait PA was positively correlated with resting RSA. Extending these findings, Kop et al. [38] used a combined design involving both a positive mood induction (Happiness Recall) and a stress task. Higher state PA during the mood induction was associated with higher HF-HRV, suggesting enhanced parasympathetic activity under emotionally congruent, non-stressful conditions.

Focusing on the stress reactivity phase, positive associations between PA and HRV remain apparent. Acevedo et al. [28] found that participants in both the low- and high-arousal PA induction conditions showed greater increases in RMSSD during a cold pressor task compared to those in the neutral control condition, indicating enhanced vagal engagement under acute stress. RMSSD reactivity was operationalized as the change from the post-induction period to the stressor period, with more positive values reflecting greater parasympathetic activation during the pain task.

During the recovery phase, divergent effects seem to emerge depending on the type of PA examined. Papousek et al. [48] revealed that higher trait PA was associated with more efficient recovery of the LF/HF ratio and LF-HRV, whereas elevated state PA corresponded with less efficient recovery of LF/HF ratio and LF-HRV. Of note, four studies [44, 46, 59, 63] did not find significant relations between HRV and PA during recovery.

Studies investigating the relation between PA and HRV in relation to real-life stress (assessed via daily questionnaires on perceived stress) reveal a comparable pattern. Martinez et al. [42] examined perceived stress and affect in daily life and reported that VLF was correlated positively with PA, while LF/HF and SDANN were negatively correlated with PA. Interestingly, Verkuli et al. [60] showed that prolonged non-metabolic reductions in RMSSD in everyday life were associated with reduced PA. Of note, Schilling et al. [52] examined feelings of stress and anger in a work context, but no significant associations between PA and RMSSD were evident.

### PA-HRV Relations in Real-Life Contexts

In everyday life settings, the relationship between PA and HRV exhibits considerable variability, reflecting diverse methodological approaches and measurement contexts. Beatton et al. [29] demonstrated that state PA was negatively associated with a higher LF/HF ratio during daily activities. Similarly, Määttänen et al. [41] reported a positive correlation between state PA and SDNN in real-life environments. In contrast, Hachenberger et al. [36], who focused on daily activities and body position, showed that state PA was positively associated with LF/HF ratio and negatively associated with HF and RMSSD.

Schwerdtfeger et al. [53] further nuanced these findings by showing that different modes of PA yielded divergent associations with RMSSD in real-life contexts. They examined both momentary PA (which reflects state PA) and aggregated PA reflecting trait-like PA. Intraindividual (within-person) as well as interindividual (between-person) effects were analysed. Findings on within-person effects of PA on HRV were heterogeneous, though coherent. Momentary deactivated PA showed a positive association with RMSSD, whereas momentary activated PA was significantly negatively related with RMSSD. This indicates that vagal activity was lower when feeling more activated and higher when feeling relaxed, thus illustrating allostatic adjustments. Interestingly, the between-person effects of PA and HRV revealed that participants with higher aggregated activated PA demonstrated elevated RMSSD, while no effects were found for aggregated deactivated PA. A separate study by Schwerdtfeger et al. [54], however, found that aggregated deactivated PA throughout the day was associated with higher nocturnal RMSSD.

Studies combining laboratory and ambulatory approaches show mixed results. Gouin et al. [35] recorded resting baseline HRV, and HRV during a worry induction protocol, and gathered PA over a 14-day period via daily diaries. Findings showed a higher RSA reactivity in relation with lower PA. However, Bylsma et al. [31], who investigated resting HRV in a controlled laboratory setting alongside daily assessments of PA, could not find significant an association between PA in daily life and HRV.

### (Sub-) Clinical Samples & Interventions

Two studies focused on samples with suspected or evident cardiovascular diseases. Bhattacharya et al. [30] reported that among patients with suspected coronary artery disease, higher PA was accompanied by greater HF and lower LF-HRV, both during the day and at night. Similarly, Lane et al. [38] observed that in patients with Long QT syndrome, both activated and low-arousal PA were associated with HF-HRV during daily activities. Low arousal PA was linked to enhanced vagal tone, while activated PA was associated with diminished vagal tone.

Three studies focused on individuals suffering from depression. Shell et al. [55] examined the link between trait PA and HF-HRV in individuals with depression. Findings did not indicate significant associations. Similarly, Fernandes et al. [34] observed no relationship between PA and HRV in antidepressant-free participants with moderate-to-severe major depressive disorder. Conrad et al. [32] investigated circadian mood variations and HRV in depressed and nondepressed volunteers at risk of cardiovascular disease. No association between PA and HRV was evident.

Intervention tools for depression and their connection to PA and HRV were examined in two studies. Petrochi et al. [49] examined state PA and HRV within the context of a depression treatment tool. Their findings indicated that increases in soothing PA (feelings of relaxation and calmness) were accompanied by increases in RMSSD. Matos et al. [43] investigated whether changes in HRV mediated the effects of a compassionate mind training (CMT) intervention on positive affect. Their findings indicate that increases in RMSSD significantly mediated the intervention’s impact on inducing relaxed PA, characterized by feelings of calmness and relaxation.

### Individual Differences and Dynamic Variability in PA-HRV Relations

Divergent findings regarding the relation between PA and HRV are also evident when examining specific subpopulations. For instance, two studies examined PA and HRV in older adults, revealing divergent results. *Kochli-Hailovski et al.* [37] reported that lower PA was associated with diminished HRV, modelled as a latent variable composed of SDNN and RMSSD, whereas Pinto et al. [50], who examined the mediating role of PA on the association between coping resources and HF-HRV, did not find any significant relation.

Two studies focused on PA instability, which describes the tendency to experience unusually large and/or frequent changes in affect. Koval et al. [39] combined laboratory HRV measurements and fluctuations of PA during the day and reported that PA instability was negatively related to both HF-HRV and RMSSD. In contrast, Rost et al. [51] measured resting HRV and connected it with daily PA assessments over two weeks, focusing on PA instability. No significant relationships between PA instability and RMSSD were found.

Weiss et al. [62] examined the relation with positive emotion dysregulation, which refers to difficulties in generating and managing the intensity and duration of positive emotions, and found positive relations between positive emotion dysregulation and resting HF-HRV at high state PA, and a negative association between positive emotion dysregulation and resting HF-HRV at low state PA.

### Patterns of PA-HRV Relations

The assumption of a strictly linear relationship between PA and HRV was tested in four studies. Duarte et al. [33] and Spangler et al. [57] investigated whether the association between PA and HRV is linear. Duarte et al. [33] uncovered a quadratic relationship between PA and HF-HRV. In addition, Spangler et al. [57] demonstrated that gender could modulate the relation between PA and HRV. While men exhibited a linear association between RMSSD and PA, a U-shaped curve best described the pattern in women. However, Sloan et al. [56] and Sveinsdóttir et al. [58] who also examined the linear relationship between PA and HRV, found no significant results.

## Discussion

The purpose of this review was to elucidate the relationship between PA and HRV in the current literature, with particular focus on variation in methodologies, study design and outcomes. Overall, the reviewed studies do not yield a consistent pattern regarding the relationship between PA and HRV. The overall picture remains ambiguous, due to variations in how PA is conceptualized, differences in study context and inconsistencies in sample sizes and examined moderators.

### Nature of the Stress Tasks

Within stress contexts, evidence suggests that the interplay between PA and HRV is complex and highly dependent on the nature of the stressor. Experimental laboratory studies analyzing the full stress induction period documented distinct associations between PA and HRV that reflect divergent autonomic responses. Specifically, individuals with high trait PA tend to show augmented cardiac vagal regulation, as indicated by elevated RMSSD during stress tasks, while momentary state PA appears to be linked with increased sympathetic tone during socially evaluative stress tasks. Differences may stem from the stress tasks employed, as socially evaluative tasks likely trigger a more pronounced sympathetic response due to fear of social judgment, whereas controlled laboratory tasks minimize evaluative threat. Notably, using verbal stress protocols to induce stress may compromise HRV indices, as speaking introduces artifacts that can distort the measurements and potentially lead to inaccurate interpretations of autonomic responses.

Further nuance emerges when examining specific stress phases. During the preparation phase, HRV measures (HF-HRV and resting RSA, respectively) were positively correlated with state PA, indicating that state PA may foster a more adaptive autonomic state before the onset of stress. In the stress response phase, higher state PA was associated with higher HF-HRV and steeper increases in RMSSD reactivity, thus highlighting dynamic autonomic adjustments during acute stress. The recovery phase presents a more complex picture. Higher trait PA was linked to more efficient recovery of both the LF/HF ratio and LF-HRV, whereas elevated state PA was associated with less efficient recovery. This divergence between state and trait PA and recovery suggests that while enduring PA may support more adaptive physiological recovery, transient positive emotions might not yield the same benefit. Of note, Papousek et al. [48] highlight that rumination might have influenced the recovery period in context with high state PA.

It is also important to highlight, that four studies did not find significant associations between state PA and cardiac vagal activity, a finding that might be attributed to the considerable variability in stressor types employed, each capable of eliciting distinct autonomic responses.

### Real-life Stress

A very heterogeneous picture is also evident when examining studies on real-life stress. Martinez et al. [40] showed that in daily life, state PA was positively associated with VLF power while being inversely related with both LF/HF ratio and SDANN. This pattern suggests that PA might be associated with a more adaptive autonomic response, which is characterized by slow regulatory processes (reflected in VLF) in connection with greater vagal activity (indicate through lower LF/HR ratio and SDNN). Verkuli’s [60] finding that prolonged reductions in RMSSD were linked to decreased explicit PA further reinforces the idea that sustained vagal tone is critical for maintaining positive emotional states during stress. Decreased parasympathetic activity under stress may signal a diminished capacity for physiological recovery, thereby reducing an individual’s ability to sustain PA. In contrast, Schilling et al. [52] did not find a significant association between PA and RMSSD within the specific work environment of police officers. The stressors inherent in police work may differ qualitatively from those encountered in everyday life or in controlled laboratory settings.

### Moderator Variables

Turning to relations of PA and HRV in real life settings, studies highlight the importance of taking confounding factors into account. Differences in the direction of the relationship between PA and LF/HF ratio were evident in two studies and might be attributable to such confounders. For instance, in contrast to Määttänen et al. [41], Hachenberger et al. [36] accounted for body position, which is known to influence HRV. In contexts where body posture is controlled, an elevated LF/HF ratio might reflect a greater sympathetic response linked to increased state PA. Posture-induced changes in HRV can obscure specific contributions of the sympathetic and parasympathetic dynamics on the LF/HF ratio, making it harder to conclude whether the ratio changes due to higher PA or other factors. This underscores the context-dependent nature of the PA-HRV relationship and highlights the challenge of accounting for confounding variables (e.g., metabolic demands), especially in real-life assessments.

### Facets of PA

Different facet of PA may show diverging associations with physiological function [64]. Feelings of being energetic and activated are likely to be accompanied by stronger physiological activation, resulting in lower HRV, due to metabolic demands of mobilization and activation. Conversely, feelings of calm and relaxation tend to boost vagal modulation, leading to higher HRV and promoting restorative processes.

In line with this perspective, Schwerdtfeger et al. [53] offer compelling evidence that the specific mode of PA measurement significantly modifies the association with cardiac vagal tone. Their findings indicate that aggregated activated PA, representing trait-like PA, is positively linked to RMSSD. Conversely, when PA is captured as activated momentary state, it is associated with lower RMSSD, suggesting that transient bursts of PA might coincide with reduced vagal modulation, perhaps due to immediate arousal-related shifts in autonomic balance, potentially indicating behavioural approach. Additionally, the observation that aggregated deactivated PA (feeling calm and relaxed) throughout the day correlates with higher nocturnal RMSSD underscores the potential restorative benefits of a more subdued, stable form of PA during recovery periods.

Importantly, the associations between PA and HRV may differ depending on the level of analysis. Between-person (interindividual) relations and within-person (intraindividual) dynamics do not necessarily point in the same direction. Making interference from one level to the other requires strict conditions (i.e., ergodicity) that are rarely met in psychological studies (70). Thus, it is essential to analyse both inter- and intraindividual associations between the various facets of PA and HRV. The results of Bylsma et al. [31] integrate into this view, reporting no link between RMSSD and aggregated (trait-like) PA ratings. Hachenberger et al. [36] found a negative link between RMSSD and HF-HRV and state-like activated PA (after controlling for body position), which was most pronounced immediately before reporting PA (≤ 10 min) and disappear when taking 30 min into account. This highlights that the timeline of HRV assessment is also important (at least for real life data). Accordingly, Schwerdtfeger and Rominger hypothesized that short-lived HRV fluctuations (within minutes) might be associated with different psychological phenomena compared to more long-lived changes [70]. Shorter periods of RMSSD directly before the prompt might therefore predict states of activated PA better than prolonged episodes [36, 53].

### A Matter of Linearity?

Furthermore, the review indicates that many studies treat the relationship between PA and HRV as linear. However, four studies have challenged this assumption. Duarte et al.‘s [31] discovery of a quadratic relationship between PA and HF-HRV implies that the benefits of PA on autonomic function may peak at moderate levels, with both low and high extremes possibly linked to less optimal HRV. This non-linear pattern is further complicated by gender differences, as Spangler et al. [55] found that while men tend to exhibit a straightforward linear association between RMSSD and PA, women display an inverted U-shaped curve, indicating that the association might differ fundamentally across sexes. The findings support the vagal tank theory [26], suggesting that moderate levels of PA are most effective in restoring vagal tone. The inverted U-shaped relationship in depressed women indicates that both low and high PA levels might impair vagal function, emphasizing the need for balance. Of note, Spangler et al. [57] examined individuals suffering from depression, a population that tends to show generally diminished PA. The limited range of PA may influence the observed non-linear relations, since higher levels of PA are less frequently observed in patients with depression.

Spangler et al. [57] assessed depressive symptoms in undergraduates using self-reports. In clinical samples, the relationship between PA and HRV often appears nonsignificant, likely due to autonomic dysregulation or limited variance. However, Petrocchi et al. [48] found that increases in soothing PA were associated with higher RMSSD, suggesting that targeted interventions can improve cardiac vagal tone. Additionally, Matos et al. [43] indicate that the ANS may be a key pathway in these effects, as their findings show that enhanced vagal tone significantly mediates the intervention’s impact on promoting a relaxed state of PA, highlighting the critical role of parasympathetic activity in emotion regulation.

While these findings underscore the complexity of the PA-HRV relationship in depression, research focusing on cardiovascular conditions offers a contrasting perspective. Higher PA was linked with markers of enhanced cardiac vagal activity in patients with suspected coronary artery disease, and higher HF-HRV in association with PA was found in patients with long QT syndrome. These findings suggest that in individuals at risk for cardiovascular events, PA might be associated with more adaptive autonomic flexibility and potentially reduce the risk of developing cardiovascular diseases. However, more studies in such populations are certainly needed to verify or falsify this assumption.

### Limitations

Several limitations of the present review need to be taken into consideration. First, the substantial heterogeneity in study designs, sample characteristics and outcome measures prevented to conduct a meta-analysis and quantify overall effect sizes. Second, the search strategy was narrowly focused on ‘positive affect’, which may have led to the exclusion of studies examining alternative operationalizations., such as ‘positive emotions’, ‘subjective well-being’, ‘positive mood’ or ‘happiness’. Additional studies examining discrete positive states or well-being concepts would likely have been identified. This could have provided a broader overview of the relation between specific positive emotions and cardiac outcomes. However, the strict search string focusing solely on PA ensured a more precise and replicable analysis, and reduced heterogeneity in operational definitions. Third, other cardiac/cardiovascular measures were not considered (e.g., cardiac output, peripheral resistance, blood pressure, baroreceptor reflex sensitivity), which could provide a broader evaluation of cardiac health consequences of PA.

### Future Research Directions and Recommendations

Future research should consider the following recommendations to ensure robust conclusions. First, trait PA offers valuable insights into an individual’s enduring emotional disposition, but is vulnerable to memory bias, as retrospective evaluations can be influenced by current mood [66]. Although many studies already incorporate ecological momentary assessment (EMA) [67, 68] to capture the dynamic nature of PA in real time, continued refinement and wider application of such methods can further minimize recall bias and enhance the understanding of both enduring level and momentary fluctuations in PA. Second, self-report measures should be complemented by valid and objective behavioural or neurophysiological indicators of positive affect. For example, genuine Duchenne smiles (detectable via video analysis or facial electromyography) represent a well-established marker of positive emotional experiences [71]. Third, given the potential confounding effects of negative affect (NA) on HRV, it is essential to control for negative affect in future research to arrive at net effects of PA. Additionally, controlling for daily mean PA and NA in ambulatory studies could be crucial. Baseline affective states may moderate the impact of momentary fluctuations on physiological measures, helping to distinguish state from trait effects. However, issues of multicollinearity and adequate sample size need to be carefully checked. Fourth, since the LF-HRV seems to be influenced by both sympathetic and parasympathetic branches, future research would benefit from focusing on well-validated indices, such as RMSSD, to facilitate more consistent comparisons and clearer interpretations of the PA-HRV relationship. Fifth, using EMA in combination with ambulatory HRV assessment might help to elaborate when PA-HRV relationships are strongest and HRV provides the highest predictive power. Sixth, exploring non-linear HRV parameters may prove valuable, as these metrics capture additional dimensions of autonomic regulation – such as system complexity and unpredictability [11] – not fully reflected by linear measures, thereby providing a more complete understanding of the PA-HRV relationship. Seventh, most included studies used cross-sectional designs, limiting causal inference. While theoretical models (e.g. Neurovisceral Integration Model [20], ) suggest that higher HRV may foster PA, the reverse direction in which PA enhances HRV via parasympathetic engagement is also plausible. Future research should investigate these temporal and causal dynamics using longitudinal and experimental designs.

## Conclusion

In summary, the current review underscores the complex relationship between PA and cardiac autonomic regulation. While several studies indicate that higher levels of PA are associated with favourable HRV patterns, such as enhanced vagal efference and a potentially adaptive autonomic profile (i.e., higher autonomic flexibility), the findings are not consistent and seem to be influenced by various factors such as stress context, within- or between-person relationships, gender or clinical status. Moreover, evidence from samples of individuals at risk for cardiovascular diseases hints that PA could be a buffer against autonomic dysfunction, which could have implications for disease progression. Future studies adopting extensive longitudinal designs that capture the dynamic interplay between PA and HRV across different contexts and applying cross-lagged analyses could elucidate causal relationships, inform tailored interventions, and ultimately enhance the capacity to leverage PA as a therapeutic target in clinical settings to promote cardiac health.

Overview of Heart Rate Variability (HRV) Metrics Reported Across Included Studies.

## Key References

- Hillebrand S, Gast KB, de Mutsert R, Swenne CA, Jukema JW, Middeldorp S, et al. Heart rate variability and first cardiovascular event in populations without known cardiovascular disease: meta-analysis and dose–response meta-regression. Europace. 2013;15:742–749.
  - Findings from this study suggest that low heart rate variability is associated with an increased risk of a first cardiovascular event in populations without known cardiovascular disease.

- Schwerdtfeger AR, Gerteis AKS. The manifold effects of positive affect on heart rate variability in everyday life: distinguishing within-person and between-person associations. Health Psychol. 2014;33:1065.
  - Findings from this study suggest, that cardiac effects of positive affect are dual-layered. Momentary changes elicit variable autonomic responses, while long-term activation signals a calming, health-promoting pattern.

## Data Availability

Data supporting the findings of this review are available within the article.
